# Aberrantly glycosylated integrin α3β1 is a unique urinary biomarker for the diagnosis of bladder cancer

**DOI:** 10.18632/aging.103297

**Published:** 2020-06-13

**Authors:** Di Jin, Ruiyun Zhang, Haige Chen, Chong Li

**Affiliations:** 1 Department of Urology, Ren Ji Hospital, School of Medicine, Shanghai Jiao Tong University, Shanghai 200127, China; 2 Core Facility for Protein Research, Institute of Biophysics, Chinese Academy of Sciences, Beijing 100101, China; 3 Beijing Jianlan Institute of Medicine, Beijing 100190, China

**Keywords:** bladder cancer, urinary biomarker, aberrantly glycosylated integrin α3β1, diagnosis

## Abstract

Bladder cancer (BC) is the most common malignancy of the urinary tract. We developed a new and ELISA kit for detecting aberrantly glycosylated integrin α3β1 (AG31) in human urine. We analysed urine samples (n=408) of patients with BC, renal cell carcinoma (RCC), prostate cancer (PC), cystitis, nephritis, and prostatitis from two centres in China. The subjects in the validation groups (n=2317) were recruited from other centres in China between July 2012 and September 2013. Receiver operating characteristic (ROC) curves were used to determine diagnostic accuracy. AG31 levels in urine samples were significantly higher in patients with BC than in any of the control subjects. Moreover, elevated levels of AG31 in urine could distinguish BC from benign inflammatory diseases. Finally, the urinary AG31 test was much more sensitive and specific than the NMP22 test. Therefore, the urinary AG31 test will provide an ideal and assay for the detection of BCs.

## INTRODUCTION

Bladder cancer (BC) is the most common malignancy of the urinary tract and ranks as the fourth most common cancer in men [1]. Tumours of the urinary bladder present either as non-muscle-invasive bladder cancer (NMIBC) or as muscle-invasive bladder cancer (MIBC). Approximately 75% of BC patients present with NMIBC at first diagnosis (Ta, T1 or tumour in situ (Tis)), and 25% present with muscle-invasive disease with a high risk of death from distant metastasis [2, 3]. Approximately 70% of NMIBCs recur, and the tumours of as many as 10% to 20% of patients will eventually progress to muscle-invasive cancer after treatment [4]. Different interventions against NMIBC change the biological and clinical behaviour of the disease [5]. Thus, early diagnosis and monitoring of the progression of BC are critical for successful treatment.

Cystoscopy and voided urine cytology are the most commonly used methods for the diagnosis and monitoring of BC recurrence and progression. Cystoscopy, the gold standard for the detection of BC, allows direct visualization and biopsy of the bladder urothelium. However, cystoscopy is invasive and relatively expensive, which limits its use. Cytologic testing of voided urine is the most commonly utilized non-invasive method for detecting BC. Voided urine cytology has good sensitivity for high-grade BC, but its sensitivity for the detection of low-grade tumours is only 4% to 31% [6]. Over the last decade, some urine-based assays, including one involving nuclear mitotic apparatus protein 22 (NMP22), a marker that has been marketed to diagnose BC, have been developed, but no markers have reached widespread use due to their low specificities [7–10]. Thus, cost-effective and non-invasive tools for the early diagnosis and lifelong surveillance of BC are urgently needed.

Integrins, a large family of cell membrane receptors, are involved in a variety of processes, including cell proliferation, migration, and cell extracellular matrix adhesion [11]. Integrin α3β1 acts as a high-affinity receptor for laminin, fibronectin, and collagen, whose interactions play critical roles in organogenesis and the maintenance of epithelial tissues [12, 13]. It has been reported that glycosylated integrin α3β1 appears in BC cells [14]. Notably, aberrant glycosylation has been implicated in the tumourigenesis of several tumour types [15, 16]. We previously generated an antibody, BCMab1, that specifically recognizes the aberrantly glycosylated integrin α3β1 (AG31) epitope on the membranes of BC cells [17]. We demonstrated that AG31 is specifically expressed in BC tissues but not in normal or other tumour tissues. AG31-mediated signalling triggers FAK activation in the tumourigenesis of BC. Furthermore, AG31 expression levels in tumour tissues are positively correlated with clinical severity and the prognosis of BC patients [17]. In this large-scale, multicentre validation study, we developed an ELISA-based assay to quantitatively detect AG31 levels in the voided urine of patients with BC or other urologic tumours. Our data show that the urinary AG31 test is a promising assay for the detection of BC.

## RESULTS

### Study groups

**Figure 1:**
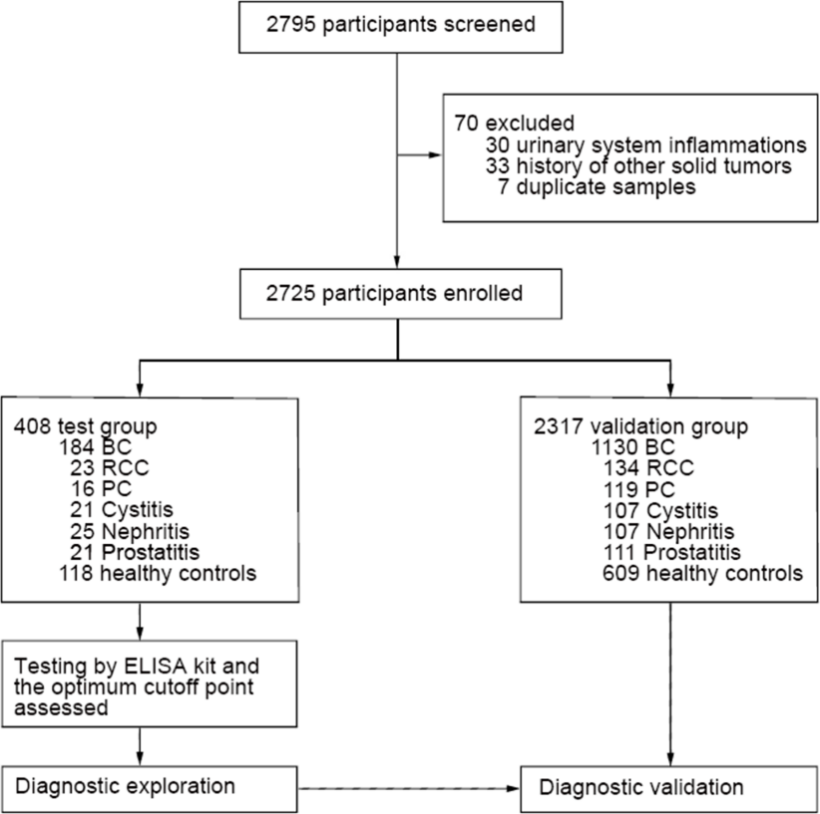
**Patient selection process and classification. Overall patient selection process and their classification based on types of diseases.**

We recruited 2725 participants overall: 408 in the test groups and 2317 in the validation groups (Figure 1). Of all the participants, 1314 had BC, and their demographic characteristics, modes of presentation, tumour stages and tumour grades are summarized in Table 1. The test population was predominantly male (78.8%), and 46.7% were over 63 years of age. Of those with a recorded presentation, 10.3% had visible haematuria, and 89.7% had non-visible haematuria. A total of 72.8% of patients had no recurrence, 14.7% experienced recurrence, and 12.5% were lost to follow-up. A total of 1130 BC patients were enrolled into the validation groups, and their clinicopathological characteristics were well matched to those of the test groups (Table 1); these two groups were well matched for age. Additionally, the patients with non-BC urologic conditions were also matched for age. Cut-off points were set as follows: 65 years of age for RCC, PC and cystitis patients, 47 years of age for nephritis patients, and 57 years of age for prostatitis patients (Supplementary Table 1).

**Table 1:** Demographic and clinicopathologic characteristics of patients with bladder cancer in the test and validation groups.

**Variable**	**Test (n=184)**			**Validation (n=1130)**			**p value**
	**No.**	**Percentage (%)**	**No.**	**Percentage (%)**			
Age (y)						
	≤63	92	50.0	490	43.4	0.078
	>63	86	46.7	609	53.9
	Missing	6	3.3	31	2.7	
Gender						
	Female	32	17.4	234	20.7	0.337
	Male	145	78.8	868	76.8
	Missing	7	3.8	28	2.5	
Pathological stage						
	Ta+ T1	134	72.8	924	81.8	0.005
	T2+T3+T4	50	27.2	206	18.2
Pathological grade					
	G1	69	37.5	279	24.7	<0.0001
	G2+G3	114	62.0	851	75.3
	Missing	1	0.5			
Hematuria					
	Visible	19	10.3	360	31.9	<0.0001
	Not-visible	165	89.7	770	68.1
Tumor recurrence						
	Yes	27	14.7	188	16.6	0.823
	No	134	72.8	887	78.5
	Missing	23	12.5	55	4.9	

### Urinary AG31 is a sensitive marker for the detection of bladder cancer

To detect AG31 levels in voided urine samples, an ELISA-based assay kit was developed by our laboratory (Supplementary Figure 1A). We generated several monoclonal antibodies that could recognize the AG31 molecule on BC tumours as previously described [17]. For establishment of the ELISA, we screened out an antibody termed BCMab3 as the capture antibody, which could strongly bind to AG31. The BCMab1 antibody also specifically recognizes AG31 on BC tumours [17], and BCMab1-conjugated horseradish peroxidase (HRP) served as the detection antibody. This ELISA kit could be used to accurately measure urinary AG31 levels (Supplementary Figure 1B). Relative light units (RLUs) were used to indicate the test levels of AG31 concentrations in urine.

**Figure 2:**
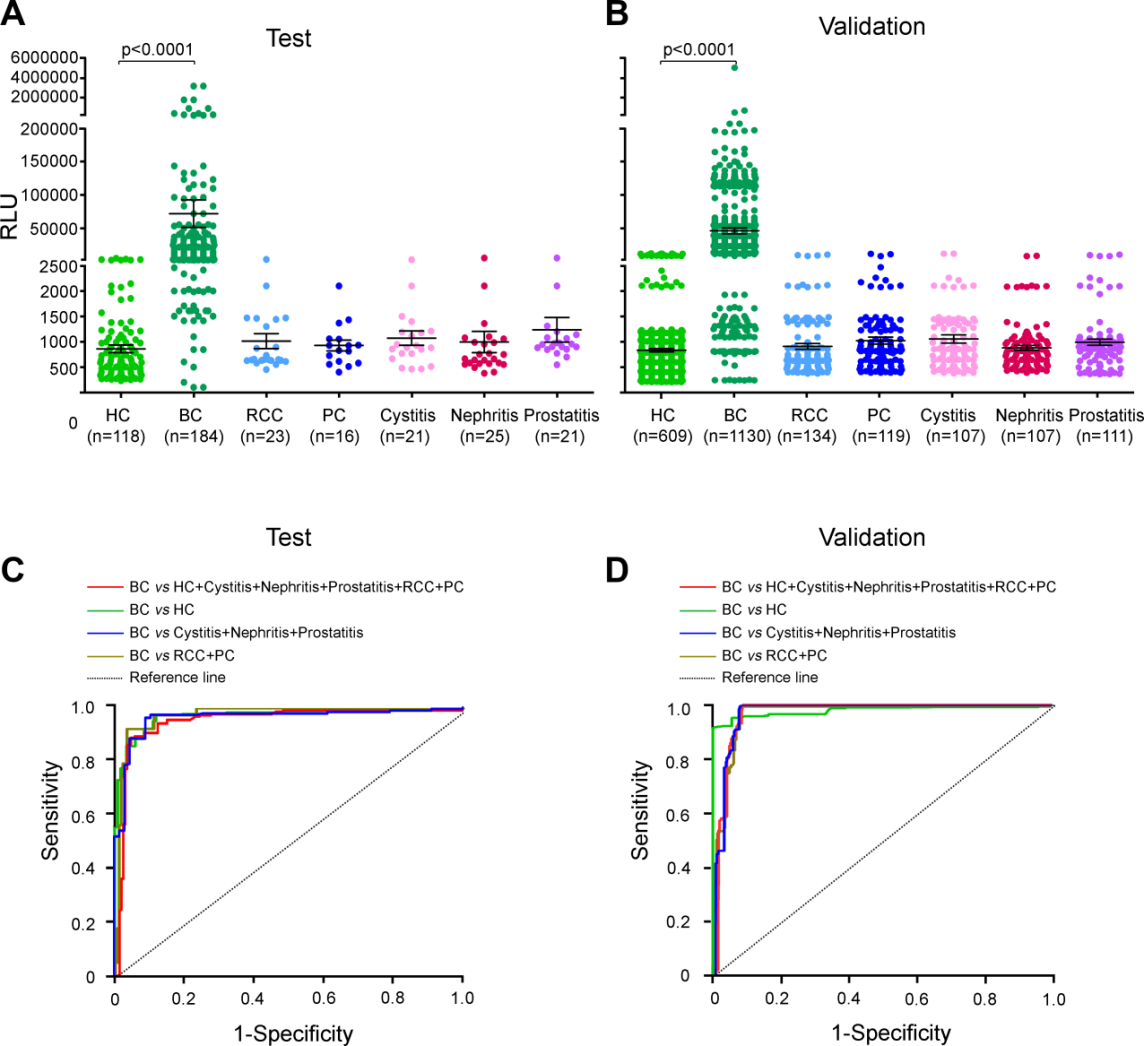
**Urinary AG31 levels are elevated in bladder cancers in the test and validation groups.** (**A**) Urinary AG31 levels for test groups. (**B**) Urinary AG31 levels for validation groups. Black horizontal lines are means, and error bars are SEs. Urinary AG31 levels were measured with RLU (relative light unit). HC, healthy control; BC, bladder cancer; RCC, renal cell carcinoma; PC, prostate cancer. (**C**) ROC curves for all patients with bladder cancer versus all controls in the test groups. (**D**) ROC curves for all patients with bladder cancer versus all controls in the validation groups. Jagged curves denote ROC curves; diagonal lines represent reference lines. HC, healthy control; BC, bladder cancer; RCC, renal cell carcinoma; PC, prostate cancer.

In the test group, urinary AG31 concentrations in the BC patients were significantly higher than those in the healthy controls (median 6525, interquartile range (IQR) 3600-26,585; mean 54,768, standard definition (SD) 276,559 [BC patients] *vs* median 576, IQR 378-988; mean 859, SD 819 [healthy controls]; p<0.0001) (Figure 2A, and Supplementary Table 2). Notably, the urinary AG31 levels in patients with RCC, PC, cystitis, nephritis, or prostatitis were comparable to those in healthy individuals (Figure 2A and Supplementary Table 2). The ROC curves were plotted for urinary AG31 in BC patients versus different test groups. For BC patients from all test groups, the area under the curve (AUC) of AG31 was 0.9567 (95% CI 0.9337-0.9797), with a sensitivity of 90.76% and specificity of 91.52% (Figure 1C, and Table 2). The optimum cut-off value was set to 1991 (Supplementary Figure 1C). The four ROC curves of AG31 levels between the BC patients and different test groups showed that the AUCs were greater than 95% (Figure 2C, and Table 2). These results were confirmed by the corresponding validation group tests (Figure 2B, 2D). Predictive values and likelihood ratios for AG31 in the diagnosis of BC are shown in Table 2. Altogether, the urinary AG31 test can distinguish BC patients from patients with other urologic tumours and benign inflammatory diseases.

**Table 2:** Urinary AG31 test for the diagnosis of bladder cancers.

	**Test**							**Validation**						
	**AUC (95% CI)**	**Sensitivity**	**Specificity**	**PPV**	**NPV**	**Positive LR**	**Negative LR**	**AUC (95% CI)**	**Sensitivity**	**Specificity**	**PPV**	**NPV**	**Positive LR**	**Negative LR**
BC *vs* HC+Cystitis+Nephritis+Prostatitis+RCC+PC												
	0.9567 (0.9337-0.9797)	0.9076	0.9152	0.8978	0.9234	10.7002	0.1010	0.9760 (0.9694-0.9826)	0.9230	0.9292	0.9255	0.9269	13.0430	0.0829
BC *vs* HC														
	0.9597 (0.9368-0.9826)	0.9076	0.9153	0.9435	0.8640	10.7098	0.1009	0.9801 (0.9741-0.9862)	0.9230	0.9442	0.9684	0.8686	16.5327	0.0815
BC *vs* Cystitis+Nephritis+Prostatitis														
	0.9500 (0.9200-0.9801)	0.9076	0.9104	0.9653	0.7821	10.1350	0.1015	0.9714 (0.9636-0.9791)	0.9230	0.9108	0.9729	0.7728	10.3441	0.0845
BC *vs* RCC+PC															
	0.9592 (0.9328-0.9856)	0.9076	0.9231	0.9824	0.6792	11.7989	0.1001	0.9720 (0.9644-0.9796)	0.9230	0.9170	0.9803	0.7273	11.1201	0.0840

HC, healthy control; BC, bladder cancer; RCC, renal cell carcinoma; PC, prostate cancer; AUC, area under curve; PPV, positive predictive value; NPV, negative predictive value; LR, likelihood ratio; CI, confidence interval.

### Urinary AG31 levels are well correlated with the clinicopathologic features of bladder cancer

**Figure 3:**
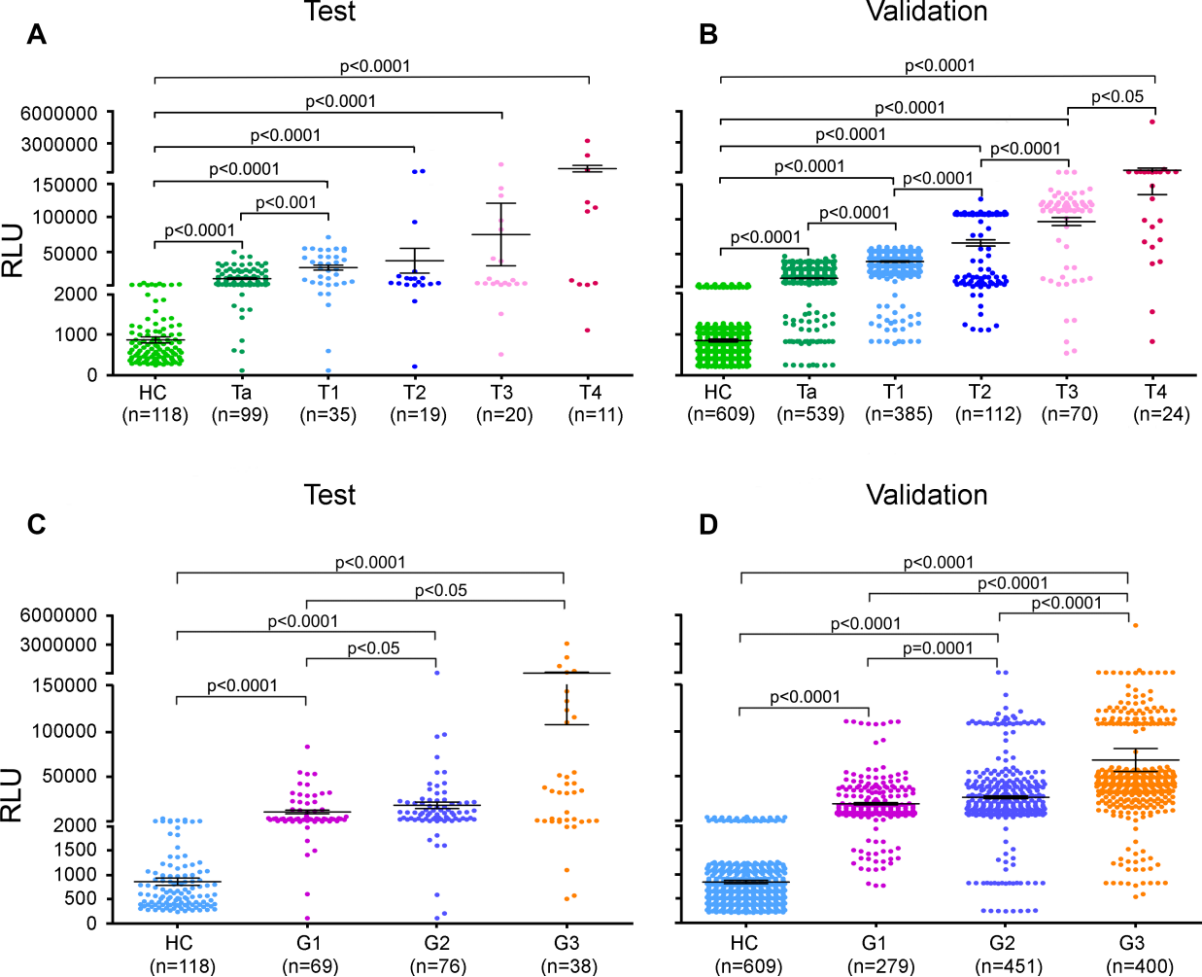
**Urinary AG31 levels distinguish between stages and grades of bladder cancer.** (**A**, **B**) Urinary AG31 levels of bladder cancer patients with different disease stages versus healthy controls in the test groups (**A**) and in the validation groups (**B**). (**C**, **D**) Urinary AG31 levels of bladder cancer patients with different grades versus healthy controls in the test groups (**C**) and in the validation groups (**D**). HC, healthy controls; RLU, relative light unit.

In the test group, the urinary AG31 levels of the BC patients with different disease stages were much higher than those of the healthy controls (Figure 3A, and Supplementary Table 2). Moreover, urinary AG31 levels were significantly increased in patients with high-stage BC. These observations were further confirmed in the validation group (Figure 3B and Supplementary Table 2). Furthermore, the AUCs for the AG31 levels of the BC patients with the different disease stages were greater than 92%, with sensitivities over 89% and specificities over 90% (Supplementary Figure 2, and Supplementary Table 3). Similarly, urinary AG31 levels increased with advancing pathological grade (Figure 3C, D, Supplementary Figure 3, Supplementary Tables 2, 3). Correlation analysis showed that urinary AG31 levels were positively correlated with tumour stage and grade (both p<0.01). Taken together, these data indicate that AG31 levels are well correlated with the clinicopathologic features of BCs.

### Urinary AG31 test is diagnostically accurate for NMIBC patients

**Figure 4:**
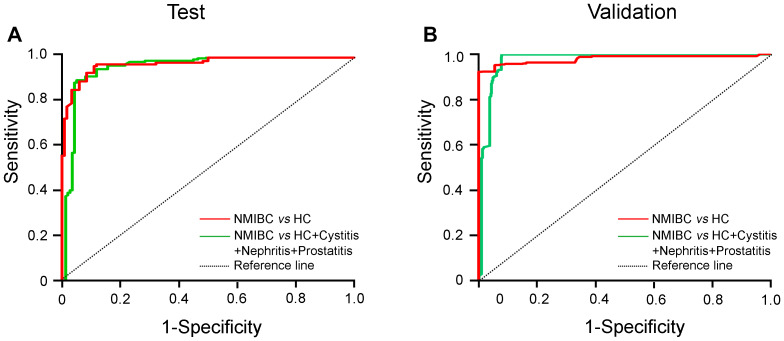
**ROC curves for urinary AG31 levels in the detection of NMIBC patients.** (**A**) ROC curves for patients with NMIBC versus the controls in the test groups. (**B**) ROC curves for patients with NMIBC versus the controls in the validation groups. Jagged curves denote ROC curves; diagonal lines represent reference lines. HC, healthy control; NMIBC, non-muscle-invasive bladder cancer.

Approximately 70% of BC patients with NMIBC (Tis+Ta+T1) will experience one or more recurrences after transurethral resection (TUR), and the BCs 10%-20% of patients will eventually progress to MIBC (3,26). Regarding recurrence and progression, patients with NMIBC are classified as low/intermediate- and high-risk groups (5,27). We next wanted to determine whether the urinary AG31 test could distinguish NMIBC patients. Of all the BC patients recruited, 1058 were classified as having stage Ta or T1 disease. The mean AG31 level of the Ta-stage patients was 13.49 times higher than that of the healthy controls in the test group (Figure 3A and Supplementary Table 2). In contrast, the mean AG31 level of the T1-stage patients was 32.11 times higher than that of the healthy controls. For the patients with NMIBC, ROC curves showed that the AUC of the AG31 levels was 0.9524 (95% CI 0.9248-0.9800), with a sensitivity of 91.04% and specificity of 91.35% in the test group compared with the patients with benign inflammatory diseases and the healthy controls (Figure 4A, and Table 3). When the NMIBC patients were compared only with the healthy controls, the AUC of the AG31 levels was 0.9574 (95% CI 0.9310-0.9838), with a sensitivity of 91.04% and specificity of 91.53% in the test group. Similar results were obtained in the validation groups (Figure 4B, and Table 3). Importantly, the diagnostic accuracy of the AG31 test was not affected by age, sex, or haematuria in BC patients (Supplementary Table 4). Altogether, these findings indicate that the urinary AG31 test is diagnostically accurate for NMIBC patients.

**Table 3:** Results for measurement of urinary AG31 in the diagnosis of non-muscle invasive bladder cancer (NMIBC) patients.

**Test**							**Validation**						
**AUC (95% CI)**	**Sensitivity**	**Specificity**	**PPV**	**NPV**	**Positive LR**	**Negative LR**	**AUC (95% CI)**	**Sensitivity**	**Specificity**	**PPV**	**NPV**	**Positive LR**	**Negative LR**
NMIBC *vs* HC															
0.9574 (0.9310- 0.9838)	0.9104	0.9153	0.9242	0.9000	10.7433	0.0978	0.9790 (0.9720-0.9860)	0.9232	0.9442	0.9617	0.8901	16.5354	0.0814
NMIBC *vs* HC+Cystitis+Nephritis+Prostatitis														
0.9524 (0.9248- 0.9800)	0.9104	0.9135	0.8841	0.9337	10.5271	0.0980	0.9758 (0.9684-0.9833)	0.9232	0.9325	0.9312	0.9246	13.6862	0.0824

HC, healthy control; NMIBC, non-muscle invasive bladder cancer; PPV, positive predictive value; NPV, negative predictive value; LR, likelihood ratio; CI, confidence interval.

### The urinary AG31 test is much more sensitive and specific than the NMP22 test

NMP22, a member of the nuclear matrix protein (NMP) family, is much more prevalent in malignant urothelial cells than in normal cells. Given that NMP22 is released into urine upon cell apoptosis, NMP22 is significantly elevated in the urine of BC patients compared to that of healthy individuals. The NMP22 test kit was approved by the U.S. Food and Drug Administration (FDA) for use in surveillance of BC. Given that the NMP22 test was used as a biomarker for the auxiliary diagnosis of BC, we applied the urinary NMP22 test and compared the results to those of the AG31 test. To further assess the diagnostic accuracy of the AG31 assay, we measured AG31 and NMP22 levels in freshly voided urine samples from BC patients (n=53) and healthy individuals (n=54). The AUC of the AG31 levels was 99.65% (95% CI: 0.9906-1.0020), with a sensitivity and specificity for the detection of BC of 90.57% and 98.15%, respectively (Supplementary Figure 4A, 4B). However, the AUC of NMP22 levels was 74.11% (95% CI: 0.6476-0.8346), and the sensitivity and specificity for the detection of BC were 47.17% and 87.04%, respectively. Discordances between the AG31 test and NMP22 test were assessed using the nonparametric McNemar test, and the *p* value was 0.01. Importantly, the predictive values and likelihood ratios for AG31 were much better than those for NMP22 (Supplementary Figure 4C). In summary, the AG31 test in urine has a better diagnostic accuracy for BC than the NMP22 test.

## DISCUSSION

We previously generated a monoclonal antibody, BCMab1, that specifically recognizes the AG31 antigen on BC tumours [17]. AG31 expression levels in tumour tissues are well correlated with the clinical severity and prognosis of BC patients. In our large-scale, multicentre study, we developed an ELISA kit based on BCMab1 and measured AG31 levels in the voided urine of BC patients. We show that urinary AG31 levels are substantially elevated in BC patients. The cut-off value was chosen to be 1991, and both the sensitivity and specificity of AG31 for the diagnosis of BC were over 90%. Moreover, the AG31 test could distinguish BC patients from healthy controls. Thus, urinary AG31 is a sensitive and specific biomarker for the detection of BC.

The diagnosis of BC currently depends on cystoscopy and urine cytology. Both examination methods have disadvantages and limitations. Cystoscopy is invasive, expensive, and associated with post-cystoscopy pain and/or risk of urinary infections [26, 27]. Cystoscopy is prone to missing flat lesions, such as those in patients with early Tis-stage BC, whereas urine cytology has a tendency to miss well-differentiated low-grade lesions [6, 28]. Furthermore, both methods rely on observer expertise, thus limiting their clinical applications. As such, there is an urgent need for a better, simpler, and cheaper diagnostic test in the diagnosis and surveillance of BC in patients. Over the last decade, several urine-based assays have been developed and become commercially available, including the NMP22, ImmnoCyt, and BTA stat tests [6, 29]. More recently, other associated protein biomarkers have been reported to be useful as urinary markers for the detection of BCs [7, 30, 31]. Given that these markers are not specific to BC, their specificities for the diagnosis of BC are usually low. Therefore, no markers have reached widespread use to date. Here, we utilized BCMab1, which recognizes the BC-specific membrane protein AG31, to develop an ELISA-based kit for the detection of AG31 released in urine.

Early diagnosis and vigilant surveillance of recurrences will immediately provide an ideal therapeutic strategy to treat BC patients. Our data show that the AG31 test has high sensitivity and specificity for detecting BCs, including early-stage BCs. Among the NMIBC patients, 638 were first diagnosed with Ta-stage BC. The AG31 test can detect Ta patients with 90.9% sensitivity and 91.5% specificity compared to healthy controls. Importantly, the AG31 test can also detect low-grade tumours with over 90% sensitivity and specificity. Thus, the urinary AG31 test is an ideal examination tool for the early diagnosis of BCs.

Due to the lack of disease-specific symptoms, the diagnosis and follow-up of BC remains a major challenge. The most common presenting symptom of BC is gross painless haematuria, usually accompanied by unexplained urinary frequency, urgency, or irritative voiding symptoms [4]. These symptoms are quite similar to those of other benign urinary infections or malignancies. To date, no biomarker has been validated for the differential diagnosis of these urinary diseases. Here, we show that the urinary AG31 test solely and specifically detects BCs but not other benign urinary infections (cystitis, nephritis or prostatitis) or other urinary malignancies, such as RCC and PC. More importantly, haematuria does not influence the detection sensitivity or specificity of AG31 for BCs.

For diagnostic evaluation of the urinary AG31 test in this study, we set the cut-off value to 1991. At this cut-off point, the sensitivity and specificity for the detection of BC are 90.76% and 91.52%, respectively. If the urinary AG31 detection kit is used for routine health screening, the cut-off point can be chosen to be 6027, in which the specificity for BC diagnosis is 100% (sensitivity is 51.63%). At this level, almost no healthy individual is predicted to have a false positive result. In contrast, if the urinary AG31 detection kit is applied to monitor therapeutic response, the cut-off point can be set to 1090, whose sensitivity for BC diagnosis is 96.74% (specificity is 77.68%). Therefore, the urinary AG31 test will serve as an ideal test tool for routine screening of BC patients.

The NMP22 test kit, approved by the FDA, is available for clinical application. Although urinary NMP22 is elevated in BC, dead and dying urothelial cells in other malignancies or inflammatory conditions can also release NMP22, thus decreasing its specificity [32, 33]. Several studies have reported that the sensitivity of NMP22 ranges from 33% to 100%, and the specificity ranges from 40% to 93% [34]. We measured NMP22 and AG31 levels in voided urine samples from BC patients and healthy controls. The sensitivity of the urinary NMP22 test is only 47.17%, which is much lower than that of the AG31 test. In conclusion, the urinary AG31 test is a sensitive and specific diagnostic test for BC that detects BCs of all stages and grades. Thus, the AG31 test will act as a promising urinary marker for the detection of BC.

## MATERIALS AND METHODS

### Primary antibodies

BCMab1 and BCMab3 monoclonal antibodies, which specifically recognize the AG31 epitope on the membranes of BC cells, were generated by our group [17]. BCMab3 was used as the capture antibody, and BCMab1 served as the detecting antibody. BCMab1 was conjugated to HRP by linkage of 2 imine-carbon (1-ethyl-3-(3-dimethylaminopropyl)-carbodiimide, EDC) as described previously [18, 19].

### Study population

From January to June 2012, we enrolled consecutive patients with BC from the Peking University People’s Hospital (Beijing, China) and Cancer Institute and Hospital, Chinese Academy of Medical Science (Beijing, China) for constitute our test group. During the same time period, we also recruited consecutive patients with RCC, prostate cancer (PC), cystitis, nephritis, or prostatitis and healthy control subjects from The First Affiliated Hospital of Shenzhen University (Shenzhen, China). From July 2012 to September 2013, validation groups comprising patients with BC, RCC, PC, cystitis, nephritis, or prostatitis and healthy control subjects were recruited from The First Affiliated Hospital of Zhejiang University (Hangzhou, China), The Second Affiliated Hospital of Kunming Medical University (Kunming, China), Renji Hospital Affiliated to Shanghai Jiaotong University School of Medicine (Shanghai, China), and The First Affiliated Hospital of Jilin University (Changchun, China).

The presence of BC was confirmed by cystoscopy, together with histopathological information obtained after subsequent surgical interventions. Tumours were graded according to the WHO criteria [20, 21], and tumour stages were defined according to the tumour-node-metastasis (TNM) staging system [22]. BC patients in this study were subdivided into five stages (Ta, T1, T2, T3, and T4) and three pathological grades (G1, G2, and G3). For the purpose of this study, we classified TNM stage Tis tumours and Ta tumours including stage Ta, Ta and T1 tumours as NMIBC. The diagnoses of cystitis and nephritis were based on symptoms, physical examination and the results of the urine culture, according to the guidelines of the Infectious Diseases Society of America and the European Society for Microbiology and Infectious Diseases [23]. The diagnosis of prostatitis was based on clinical, laboratory, and imaging evidence (X-ray, ultrasonography, CT, or MRI) [24, 25]. The presence of RCC was confirmed by symptoms, imaging studies (CT or MRI), laboratory data, and renal tumour biopsy. The diagnosis of PC was based on digital rectal examination, the prostate-specific antigen test, imaging evidence (transrectal ultrasonography, CT, MRI, or ECT), prostate biopsy, and pathohistological examination of radical prostatectomy specimens, according to the clinical guidelines of the European Association of Urology. The healthy control subjects were eligible volunteers with no diseases of the urinary system, no viral hepatitis and no malignant diseases. Individuals who had a history of other solid tumours were excluded from the study.

For the test groups, we enrolled patients with BC, RCC, PC, cystitis, nephritis, or prostatitis, along with healthy control subjects, from January to June 2012. For the validation groups, patients with BC, RCC, PC, cystitis, nephritis, or prostatitis and healthy control subjects were recruited from July 2012 to September 2013. These two groups were matched for age (≤63 years versus >63 years) and sex for comparisons of AG31 values. In this study, all urine samples of the patients with BC, RCC, PC, cystitis, nephritis or prostatitis were collected before treatment. Data collection and analysis were performed by three independent researchers (YD, CL, and QZ). Approval for the study was obtained from the institutional ethics review committee of each study centre. Written informed consent was obtained from all participants, according to the committees’ regulations.

### Measurement of urine samples for the AG31 test

Each well of a 96-well Nunc-Immuno microtiter plate with a MaxiSorp surface (Nalge Nunc, Penfield, NY, USA) was coated with 100 μL of a 4 μg/mL solution of BCMab3 in 50 mM carbonate buffer (pH 9.5), and plates were incubated at room temperature overnight. The wells were then blocked with 120 μL of 10 mM phosphate buffer (pH 7.4) containing 1% bovine serum albumin and 1% gelatine at 37 °C for 2 h. The plates were washed four times with PBS containing 0.05% Tween-20 and then dried at room temperature. These plates were kept at 4 °C. For the urinary AG31 test, 50 μL of urine samples and 50 μL of HRP-BCMab1 (1 ng/mL) were added to each well and incubated at 37 °C for 1 h. After the plates were completely washed, they were dried at room temperature. A total of 100 μL of freshly prepared substrate solution was added to each well and stirred. Chemiluminescence intensity was measured with a chemiluminescence apparatus (Hamamatsu Photonics, Beijing, China).

### NMP22 measurement

A commercial ELISA kit (Alere Scarborough Inc., Scarborough, ME, USA) was used for auxiliary diagnosis of BC as a biomarker. According to the manufacturer’s protocol for the NMP22 test, urinary NMP22 levels were measured for comparison.

### Statistical analysis

Statistical analyses were conducted with SPSS for Windows (version 16.0) and GraphPad Prism statistical software (version 5.01). The Mann-Whitney U test was used to assess differences between two independent groups, such as the levels of urinary AG31 between the patients with BC and the healthy controls. The differences in clinicopathological stages or grades were assessed using the Kruskal-Wallis H test, which was used to analyse multiple variables. Receiver operating characteristic (ROC) analysis was used to characterize marker performance, and ROC curves were constructed to assess sensitivity, specificity, and the respective areas under the curve (AUCs) with 95% confidence intervals. In the ROC curves, the true positive rate (sensitivity) is plotted against the false positive rate (1-specificity) for different cut-off points of a parameter. We chose the optimum cut-off value for diagnosis by maximizing the sum of sensitivity and specificity and minimizing the overall error (square root of the sum [1-sensitivity]^2^ + [1-specificity]^2^) and by minimizing the distance of the cut-off value to the top-left corner of the ROC curve. The correlations between the levels of AG31 in urine and clinicopathological characteristics were analysed using Pearson’s chi-square test (χ^2^) or Fisher’s exact test. The bivariate correlation and the Spearman correlation coefficient were used to assess the magnitude of the correlation between urinary AG31 levels and tumour stages or grades. Discordances between the AG31 test and NMP22 test were assessed using the nonparametric McNemar test. In all statistical analyses, a *p* value of 0.05 or less was considered statistically significant.

## Supplementary Material

Supplementary Figues

Supplementary Tables
