# Supplementary material for: Aberrantly glycosylated integrin α3β1 is a unique urinary biomarker for the diagnosis of bladder cancer
Source: Aging (Albany NY). 2020 Jun 13;12(11):10844–62. doi: 10.18632/aging.103297 (PMC7346044; doi:10.18632/aging.103297)
Supplement: Supplementary Figues [file aging-12-103297-s002..pdf]

SUPPLEMENTARY FIGURES

A

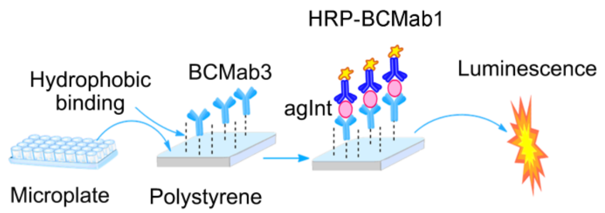

B

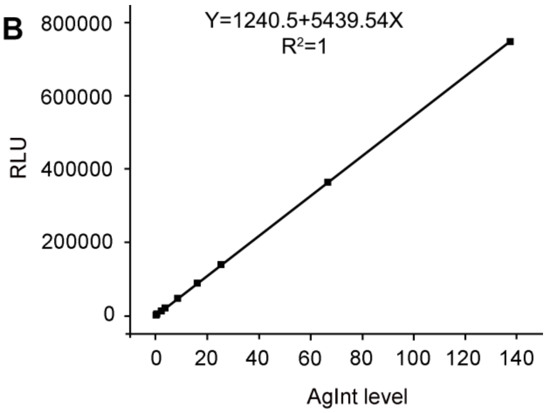

C

Test	%Sensitivity(95% CI)	%Specificity (95% CI)
≥1090 cutoff point	96.74 (93.04 to 98.79)	77.68 (71.65 to 82.96)
≥ 1991 cutoff point	90.76 (85.62 to 94.53)	91.52 (87.07 to 94.82)
≥ 6027 cutoff point	51.63 (44.16 to 59.04)	100.0 (98.37 to 100.0)

Supplementary Figure 1. Development of the urinary AG31 test kit. (**A**) Strategy of the ELISA-based AG31 test assay. (**B**) Calibration curve of the AG31 test with the standards. AG31: aberrantly glycosylated integrin α3β1; BCMab1: one monoclonal antibody against AG31; BCMab3: another monoclonal antibody against AG31; HRP: horseradish peroxidase; RLU: relative light unit.

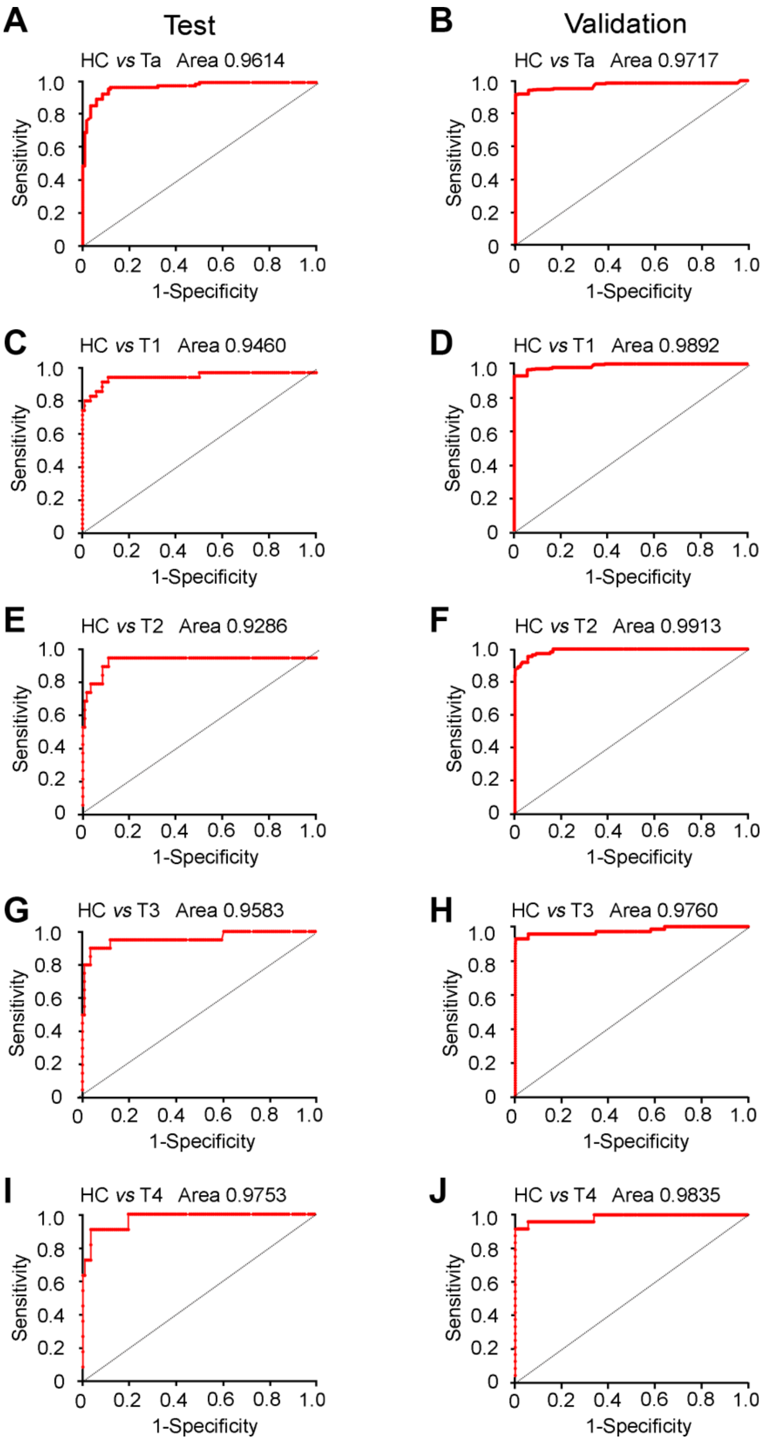

Supplementary Figure 2. Diagnostic capabilities of urinary AG31 test in discriminating bladder cancer patients with different stages versus healthy controls. (**A**, **B**) Receiver operating characteristic (ROC) curve for patients with Ta stage versus healthy controls in the test group (**A**), and in the validation group (**B**). (**C**, **D**) ROC curve for patients with T1 stage versus healthy controls in the test group (**C**), and in the validation group (**D**). (**E**, **F**) ROC curve for patients with T2 stage versus healthy controls in the test group (**E**), and in the validation group (**F**). (G, H) ROC curve for patients with T3 stage versus healthy controls in the test group (**G**), and in the validation group (**H**). (**I**, **J**) ROC curve for patients with T4 stage versus healthy controls in the test group (**I**), and in the validation group (**J**). A jagged curve denotes a ROC curve; a diagonal line represents a reference line. HC, healthy controls.

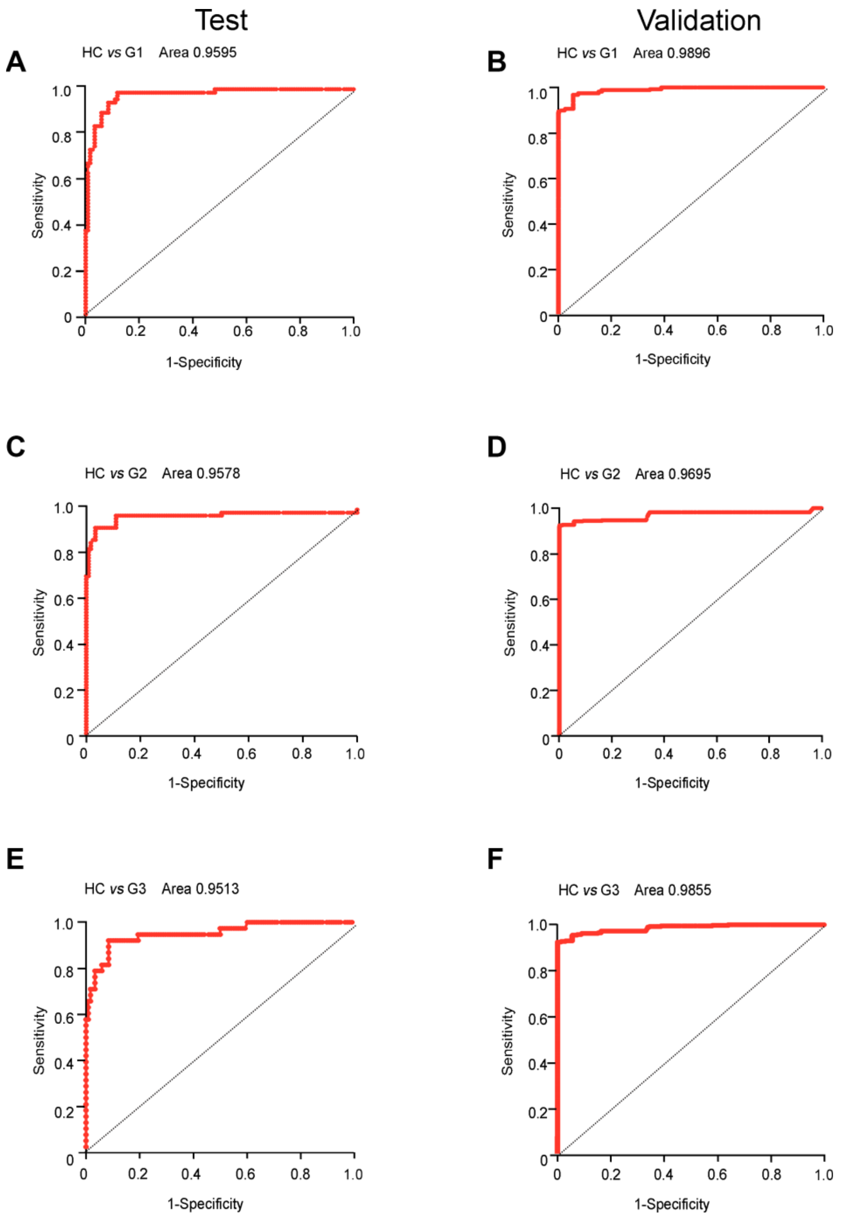

Supplementary Figure 3. Diagnostic capabilities of urinary AG31 test in discriminating bladder cancer patients with different grades versus healthy controls. (**A**, **B**) Receiver operating characteristics (ROC) curve for BC patients with G1 versus healthy controls in the test group (**A**), and in the validation group (**B**). (**C**, **D**) ROC curve for BC patients with G2 versus healthy controls in the test group (**C**), and in the validation group (**D**). (**E**, **F**) ROC curve for BC patients with G3 versus healthy controls in the test group (**E**), and in the validation group (**F**). A jagged curve denotes a ROC curve; a diagonal line represents a reference line. HC, healthy controls.

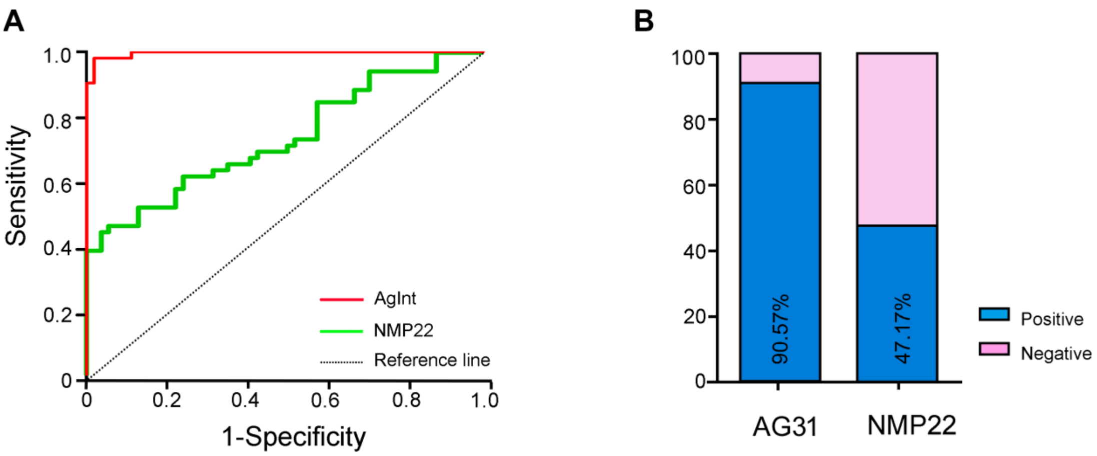

C

	AUC (95% CI)	Sensitivity (%)	Specificity (%)	PPV (%)	NPV (%)	Positive LR	Negative LR	p value
**AG31 Test**								
	0.9965 (0.9906-1.0020)	0.9057	0.9815	0.9796	0.9138	48.9057	0.0961	0.01
**NMP22 Test**								
	0.7411 (0.6476-0.8346)	0.4717	0.8704	0.7813	0.6267	3.6388	0.6070	

AUC, area under curve; PPV, positive predictive value; NPV, negative predictive value; LR, likelihood ratio; CI, confidence interval.

Supplementary Figure 4. The urinary AG31 test is more sensitive and specific than the NMP22 test. (**A**) ROC curves for AG31 and NMP22 for the patients with bladder cancer versus healthy controls in the test group. Jagged curves denote ROC curves; the diagonal line represents a reference line. (**B**) The rates of positive results for AG31 or NMP22 in all tested bladder cancer patients in the test group. (**C**) Comparison of the AG31 test with NMP22 test for the diagnosis of bladder cancer.
