# Supplementary material for: Aberrantly glycosylated integrin α3β1 is a unique urinary biomarker for the diagnosis of bladder cancer
Source: Aging (Albany NY). 2020 Jun 13;12(11):10844–62. doi: 10.18632/aging.103297 (PMC7346044; doi:10.18632/aging.103297)
Supplement: Supplementary Tables [file aging-12-103297-s001..pdf]

SUPPLEMENTARY TABLES

Supplementary Table 1A. Age distribution of patients with renal cell carcinoma (RCC) in the test and validation groups.

Variable		Test (n=23)		Validation (n=134)		p value
		No.	Percentage (%)	No.	Percentage (%)	
Age (years)						
	≤65	14	60.9	66	49.3	0.303
	>65	9	39.1	68	50.7	

Supplementary Table 1B. Age distribution of patients with prostate cancer (PC) in the test and validation groups.

Variable		Test (n=16)		Validation (n=119)		p value
		No.	Percentage (%)	No.	Percentage (%)	
Age (years)						
	≤65	12	75.0	65	54.6	0.122
	>65	4	25.0	54	45.4	

Supplementary Table 1C. Age distribution of patients with cystitis in the test and validation groups.

Variable		Test (n=21)		Validation (n=107)		p value
		No.	Percentage (%)	No.	Percentage (%)	
Age (years)						
	≤65	9	42.9	56	52.3	0.427
	>65	12	57.1	51	47.7	

Supplementary Table 1D. Age distribution of patients with nephritis in the test and validation groups.

Variable		Test (n=25)		Validation (n=107)		p value
		No.	Percentage (%)	No.	Percentage (%)	
Age (years)						
	≤47	12	48.0	56	52.3	0.696
	>47	13	52.0	51	47.7	

Supplementary Table 1E. Age distribution of patients with prostatitis in the test and validation groups.

Variable		Test (n=21)		Validation (n=111)		p value
		No.	Percentage (%)	No.	Percentage (%)	
Age (years)						
	≤57	10	47.6	59	53.2	0.642
	>57	11	52.4	52	46.8	

Supplementary Table 2. Urinary AG31 levels in different measurement groups in both test and validation groups.

Group	Test					Validation				
	No.	Median (IQR)	Mean (SD)	times	p value	No.	Median (IQR)	Mean (SD)	times	p value
HC	118	576 (378-988)	859 (819)			609	660 (440-909)	840 (858)		
BC	184	6525 (3600-26585)	54768 (276559)			1130	23342 (11459-45637)	40281 (150259)		
RCC	23	648 (630-1440)	1011 (703)			134	667 (601-902)	916 (664)		
PC	16	875 (586-1054)	925 (427)			119	844 (611-1240)	1031 (777)		
Cystitis	21	885 (715-1201)	1070 (652)			107	863 (634-1375)	1064 (846)		
Nephritis	25	663 (569-1022)	995 (1048)			107	755 (560-990)	886 (494)		
Prostatitis	21	955 (866-1109)	1234 (1114)			111	914 (702-1045)	998 (656)		
Ta	99	5409 (3487-19089)	11584 (11522)	13.49		539.	12262 (10593-17900)	15250 (9247)	18.15	
T1	35	26860 (5326-43584)	27585 (21465)	32.11		385.	42489 (32469-50096)	39146 (14676)	46.60	
T2	19	6609 (2814-15289)	37709 (78583)	43.90	0.008	112.	106507 (10817-107541)	65989 (47413)	78.56	<0.001
T3	20	5933 (4183-72474)	75671 (203268)	88.09		70.	114721 (84494-124044)	96689 (47844)	115.11	
T4	11	109344 (3340-411814)	521252 (1022000)	606.81		24.	155164 (68320-189943)	336146 (982861)	400.17	
G1	69	5115 (3278-15313)	12667 (16439)	14.75		279	12153 (10437-27527)	20825 (20877)	24.79	
G2	76	11611 (4740-22854)	19828 (30457)	23.08	0.010	451	16621 (11065-35386)	27772 (29740)	33.06	<0.001
G3	38	33621 (2845-110717)	202477 (589592)	235.71		400	44916 (32455-56397)	67956 (247740)	80.90	

HC, healthy controls; BC, bladder cancer; RCC, renal cell carcinoma; PC, prostate cancer; IQR, interquartile range; SD, standard definition.

Supplementary Table 3. Results for measurement of urinary AG31 in the diagnosis of bladder cancer with different stages and grades *versus* healthy controls.

	Test							Validation						
	AUC (95% CI)	Sensitivity	Specificity	PPV	NPV	Positive LR	Negative LR	AUC (95% CI)	Sensitivity	Specificity	PPV	NPV	Positive LR	Negative LR
HC *vs* Ta														
	0.9614 (0.9341-0.9887)	0.9091	0.9153	0.9000	0.9231	10.7273	0.0993	0.9717 (0.9606-0.9829)	0.9184	0.9442	0.9357	0.9289	16.4496	0.0865
HC *vs* T1														
	0.9460 (0.8846-1.0070)	0.9143	0.9153	0.7619	0.9730	10.7886	0.0937	0.9892 (0.9838-0.9945)	0.9299	0.9442	0.9133	0.9551	16.6556	0.0743
HC *vs* T2														
	0.9286 (0.8283-1.0290)	0.8947	0.9153	0.6296	0.9818	10.5579	0.1150	0.9913 (0.9855-0.9971)	0.9196	0.9442	0.7518	0.9846	16.4724	0.0851
HC *vs* T3														
	0.9583 (0.9000-1.0170)	0.9500	0.9153	0.6552	0.9908	11.2100	0.0546	0.9760 (0.9504-1.0020)	0.9286	0.9442	0.6566	0.9914	16.6324	0.0757
HC *vs* T4														
	0.9753 (0.9401-1.0110)	0.9091	0.9153	0.5000	0.9908	10.7273	0.0993	0.9835 (0.9561-1.0110)	0.9167	0.9442	0.3929	0.9965	16.4191	0.0883
HC *vs* G1														
	0.9595 (0.9254-0.9936)	0.9130	0.9153	0.8630	0.9474	10.7739	0.0950	0.9896 (0.9840-0.9951)	0.9068	0.9442	0.8815	0.9567	16.2426	0.0987
HC *vs* G2														
	0.9578 (0.9192-0.9964)	0.9079	0.9153	0.8734	0.9391	10.7132	0.1006	0.9695 (0.9565-0.9826)	0.9268	0.9442	0.9248	0.9457	16.6011	0.0775
HC *vs* G3														
	0.9513 (0.9093-0.9933)	0.9211	0.9153	0.7778	0.9730	10.8684	0.0863	0.9855 (0.9786-0.9924)	0.9300	0.9442	0.9163	0.9536	16.6579	0.0741

HC, healthy control; AUC, area under curve; PPV, positive predictive value; NPV, negative predictive value; LR, likelihood ratio; CI, confidence interval.

Supplementary Table 4. Correlation between AG31 levels and clinicopathologic characteristics of bladder cancer patients in both test and validation groups.

Variable		Test (n=184)			Validation (n=1130)		
		Negative (Percentage)	Positive (Percentage)	p value	Negative (Percentage)	Positive (Percentage)	p value
		17	167		87	1043	
Age (years)							
	≤63	9 (9.78%)	83 (90.22%)	0.702	31 (6.33%)	459 (93.67%)	0.787
	>63	7 (8.14%)	79 (91.86%)		41 (6.73%)	568 (93.27%)	
	Missing	1	5		15	16	
Gender							
	Female	4 (12.50%)	28 (87.50%)	0.495+	13 (5.56%)	221 (94.44%)	0.425
	Male	12 (8.28%)	133 (91.72%)		61 (7.03%)	807 (92.97%)	
	Missing	1	6		13	15	
Pathological stage							
	Ta+T1	12 (8.96%)	122 (91.04%)	0.718+	71 (7.68%)	853 (92.32%)	0.968
	T2+T3+T4	5 (10.00%)	45 (90.00%)		16 (7.77%)	190 (92.23%)	
Pathological grade							
	G1	6 (8.70%)	63 (91.30%)	0.986	26 (9.32%)	253 (90.68%)	0.242
	G2+G3	10 (8.77%)	104 (91.23%)		61 (7.17%)	790 (92.83%)	
	Missing	1					
Hematuria							
	Visible	4 (21.05%)	15 (78.95%)	0.081+	26 (7.22%)	334 (92.78%)	0.681
	Non-visible	13 (7.88%)	152 (92.12%)		61 (7.92%)	709 (92.08%)	
Tumor recurrence							
	Yes	2 (7.41%)	25 (92.59%)	1.000+	23 (12.23%)	165 (87.77%)	<0.001
	No	13 (9.70%)	121 (90.30%)		44 (4.96%)	843 (95.04%)	
	Missing	2	21		20	35	

+: Fisher exact test. Chi-square tests for all the other analysis.
